# The training and development needs of midwives in Indonesia: paper 2 of 3

**DOI:** 10.1186/1478-4491-4-9

**Published:** 2006-04-19

**Authors:** Deborah Hennessy, Carolyn Hicks, Harni Koesno

**Affiliations:** 1 School of Health Sciences, University of Birmingham, Birmingham, UK; 2 Indonesian Midwives Association, Jakarta, Indonesia

## Abstract

**Background:**

There is a shortfall in midwives in Indonesia (an estimated 26 per 100 000 people), which means that the quality of antenatal, perinatal and postnatal care varies widely. One consequence of this is the high rate of maternal and perinatal mortality, which has prompted a number of health initiatives. The current study was part of a review of the existing complex system of midwifery training and the development of a coherent programme of continuing professional development, tighter accreditation regulations and clearer professional roles. Its aims were to identify the occupational profiles and development needs of the participating midwives, and to establish whether any differences existed between grades, geographical location and hospital/community midwives.

**Methods:**

A psychometrically valid training-needs instrument was administered to 332 midwives from three provinces, covering both hospital and community staff and a range of midwifery grades. The instrument had the capacity to identify occupational roles and education/training needs of the respondents.

**Results:**

The occupational roles of the midwives varied significantly by province, indicating regional service delivery distinctions, but very little difference in the roles of hospital and community midwives. The most educated midwives attributed more importance to 35 out of the 40 tasks, suggesting an implicit role distinction in terms of level of activity. All midwives reported significant training needs for all 40 tasks. The most-educated midwives recorded training needs for 24 tasks, while the less-educated had training requirements for all tasks, which suggests that new training programmes are effective. Few differences in training needs were revealed between hospital and community midwives

**Conclusion:**

The results from this survey suggest important regional differences in how the midwife's role is discharged and underline the importance of this sort of research, in order to ensure the suitability of basic and postbasic educational provision. The study also highlights the need for further development and training of midwives in a wide range of tasks. These results provide a systematic and reliable overview of current midwifery roles and development needs and could serve to inform future training.

## Background

### Context

Midwifery in Indonesia plays a particular role in improving community, maternal and neonatal health, as well as contributing towards the health targets set by the Ministry of Health. However, despite their acknowledged importance, in 2001/2002 there were still only 26 midwives per 100 000 of the population, which in absolute and relative terms represents a severe shortfall [1].

One consequence of this is the high maternal and perinatal mortality rates, the former being estimated at 307 per 100 000 live births in 2003. Infant mortality rates for 2001 ranged from 51/1000 live births [2], to 46/1000 live births [3], while the under-five death rate is estimated to be 68/1000 live births [2].

Initiatives such as the Safe Motherhood legislation and Millennium Development Goals have agreed upon a set of targets in Indonesia, one of which is to reduce maternal deaths to 125 per 100 000 births by the year 2010. This, though, represents a national average; within Indonesia there are great inequalities of health status, with infant mortality rates ranging from 18 to 88 per 1000 live births [4].

### Inequalities in midwifery provision in Indonesia

One contributory factor to these statistics is the level of health care provision, which varies widely: in 1999, 94.3 % of births in Jakarta were attended by a health care professional, compared with 30.6% in North Lampung District, Sumatra [3]. To achieve reductions in maternal mortality, it is essential that the number of births attended by trained midwives (skilled birth attendants) is increased, and that antenatal provision is extended and made more accessible. The Ministry of Health's targets, outlined in its "Healthy Indonesia 2010" policy, specify an ambitious ratio of one midwife per 1000 of the population. Recent data indicate that wide variations in midwifery provision exist, from one midwife per 1.5 villages in Nanggroe Aceh Darussalem to one per 35 villages in the province of Maluku [1]. Furthermore, the population of villages varies from 3000 to 10 000 and the distances between them may be considerable, further hampering the delivery of midwifery care. The health demands, facilities, support and supervision mechanisms available within these contexts differ greatly and place divergent demands on the midwives.

### The education and training of midwives in Indonesia

To assist deliveries the community looks to traditional birth attendants (or Dukuns), who, despite limited training and a low skills base, may be responsible for an estimated 34% of deliveries. This is in direct contravention of the Midwifery Ministerial Decree of 2000, which stipulated that only trained midwives be responsible for deliveries. The basic education, training and skill base of the Dukuns are well below the national standard of the trained Bidan Desa (also known as village midwife).

Further midwifery support for remote rural communities is provided by the Bidan Desa programme, consisting of a one-year basic programme for graduates of a junior high school nursing programme (which leads to a health certificate). Starting in 1989 and finishing in 1996, when it was replaced by a three-year post-high school Diploma Programme (D3) for midwives, the programme trained 59 000 midwives for rural practice.

However, those midwives trained under the system require regular updating, in order to enhance their skill base. Data collected in 2001 suggested that over 90% of nurses and midwives sampled had had no postbasic or continuing professional development (CPD) training in the past three years, which in a global era of rapid health care developments means that much midwifery care in Indonesia may not be conducted according to current evidence-based best practice [5]. This synopsis demonstrates the current significant shortfall in provision, some of which may be suboptimal, and confirms the need to train and develop increasing numbers of competent midwives.

### The impact of the absence of job descriptions for midwives

Despite these apparent differences in preparation, the two midwifery grades do not have commensurate local job descriptions attached to them, which means there is no formalized record of what they are required to do, the standards to which they should be working and to whom they should be accountable. For example, a preliminary survey conducted by the Ministry of Health with technical support from the World Health Organization [5], found that one consequence of having no job description was a misuse and underuse of midwives' skills; for example, around 60% of the midwives surveyed routinely undertook basic cleaning activities. Such misuse of resources could not be identified through the mandatory annual individual performance assessments, as these are not systematically or objectively conducted with midwives and because of resource and reviewer training issues. In addition, the absence of locally produced job descriptions makes it difficult to monitor and record individual practice and development needs.

The situation is further exacerbated by the inconsistent dissemination of midwifery standards to practitioners. Despite a concerted effort by the Ministry of Health and the Midwifery Professional Association, the national midwifery standards published by the Ministry of Health in 2001 have been distributed to hospital and community health centres in only 24 out of 34 provinces. Consequently, there is great variability in the standards of clinical service, which together with the diversity of basic educational provision and lack of resources, has meant that midwifery care is often something of a lottery.

### The role of competence-based training for midwives

In 1997, in-service competence-based training was introduced to teach core clinical competences (such as care for normal birth, basic emergency obstetric and neonatal care and post-abortion care), to maternity care health providers, to support the Making Pregnancy Safer initiatives. While a recent review of this major initiative highlighted its successes, a number of areas requiring further development were identified [5].

The recommendations emerging from this review included: an imperative that the quality of competence-based training should be monitored and controlled; that course content and delivery should meet both service requirements and the realities of service provision; that the methods of delivery should be based on needs; and that training should be adapted to meet these needs. The review concluded that future competence-based training should be funded only after a specific needs assessment, with decisions made about in-service training as part of a comprehensive district human resource development plan.

### The need for a review of midwifery skills

It follows then, that a systematic review of the skills of midwives must be a rational precursor to the development of any educational programme. Moreover, if a reliable occupational profile of midwives could be obtained, then there would be a sound foundation on which to identify and define the core competences, which would form the basis of basic midwifery education in a variety of local contexts. These could be further used to inform national and regional education curricula and care standards, to accredit courses and to establish safe levels of clinical practice. They could also be the basis of a systematic programme of individual performance assessments or even Group Performance Reviews, with clear performance indicators and expected outcomes.

For those midwives already qualified, it is similarly essential to identify skill deficits and underperformance in their existing roles, so that continuing professional development can be devised that would directly address areas of suboptimal clinical performance. A systematic survey of this type would enable limited available budgets to be focused directly on areas of greatest need, thereby rationalizing resources.

### The aims of the present study

To this end the current survey was undertaken, with the following aims:

• to establish the occupational profile of midwives working in a variety of contexts, thereby informing job descriptions and basic curriculum development;

• to identify and prioritize training requirements of practising midwives, in order that there can be a focused systematic in-service education programme;

• to establish the relevance of a formal job description to midwives' role perceptions/occupational profile;

• to establish whether formal performance reviews changed self-perceived performance standards and reported training needs.

It should be noted that this paper reports findings from a larger survey undertaken in 2001, which could inform the planning of midwifery education and services. Preliminary information from the survey has been used to develop services.

## Methods

### Sample

Three hundred and thirty-two (332) midwives participated in the study. All were female and all were clinical practitioners rather than managers. Of the 332, 158 (47.6%) worked in hospitals and 174 (52.4%) worked in the community. There were 52 D3 midwives (three-year diploma in midwifery obtained from a midwifery academy or polytechnic) and 280 PPB/A midwives (one-year midwifery education, usually following a three-year nursing certificate; also known as village midwives), representing three provinces, including North Sulawesi (n = 81), East Kalimantan (n = 97) and North Sumatra (n = 154).

The provinces were selected according to specific criteria to cover developed and underdeveloped, metropolitan and rural, densely and sparsely populated regions as well as geographical variations. North Sumatra is in the West of Indonesia and is the most developed; East Kalimantan has the sparsest population and is the least developed; and North Sulawesi is the furthest east and has a geographical boundary covering a number of small, populated islands. Two districts were chosen in each of the three provinces (six districts in all), one in the main city and one in a more rural location (within two-hour access of the city). Each of the six districts was asked to identify 25 PPB/A participants from the community and 25 from hospitals. Only one district had D3 midwives and they were similarly asked to gather 25 from hospitals and 25 from community settings. The samples were quota/opportunistic. Asking the districts to find random samples was not feasible because of logistics, particularly with regard to problems for patient care when removing a large number of key practitioners from the field.

### Materials

The questionnaire described in paper 1 [6] was administered to the midwives for self-completion. The instrument comprised a biographical section and a 40-item questionnaire, which related to core activities within the midwifery role. Each item had to be rated twice along a seven-point scale. The first rating (rating A) provided an assessment of how important the task was to the successful performance of the respondent's role and provided an occupational profile; the second rating (rating B) provided a self-evaluation of how well the task was currently being performed; a comparison of the two ratings demonstrated the degree of training need for each task, in that high importance ratings on a task, coupled with low performance ratings, indicate a training need.

### Procedure

The midwives were transported by bus and boat to centres in each district, where they completed the questionnaires under the supervision of a team of data collectors. This team comprised one international or national consultant, two or three members of the working group (detailed in paper 1 [6]), a provincial coordinator from the midwifery association and five senior midwives, who were either managers or academics. This team had been trained in the use of the questionnaire, and were also able to clarify the instructions and any items in the instrument over which respondents had difficulty. The data from the completed questionnaires were entered into an SPSS database for analysis.

## Results

### The occupational role/profile of midwives

#### Whole sample

All 40 items in the questionnaire were rated as important to the midwives' role (rating A: criticality), with a range of 5.60–6.73. This suggests that all 40 tasks are essential to the midwife's role.

The criticality ratings were also subjected to exploratory factor analysis, in order to establish whether there were any coherent sets of related skills underpinning the midwife's role that could form the basis of a set of modules for a core midwifery curriculum. Standard procedures were followed for this factor analysis [7]. Using a Varimax rotation and with the KMO test of sampling adequacy = 0.937, Bartlett's test of sphericity p = 0.000, and eigen values in excess of 1.0, three factors emerged, which together accounted for 48.9% of the variance. These are presented in Table 1 (factor loadings in brackets):

**Table 1:** Factor structure of crucial midwifery competences

**Factor 1 (39.5% of the variance, Cronbach's α = 0.9324): Clinical and service management**
Instructing/training students/junior staff (0.770)
Collecting own clinical/patient/surveillance data (0.738)
Locating/accessing equipment for clinical work (0.732)
Undertaking clinical examinations of patients (0.729)
Interpreting results from clinical investigations (0.692)
Inputting data into written/computerized records (0.681)
Undertaking budget planning activities (0.680)
Actively assisting in change management activities (0.663)
Planning patients' discharge (0.653)
Introducing new ideas into own clinical work ((0.644)
Making decisions about patients' clinical problems (0.641)
Undertaking health promotion/prevention activities (0.627)
Writing clinical/shift and other reports (0.618)
Using technical equipment (0.609)
Prioritizing work according to patients' needs (0.569)
Showing patients and their families how to do things (0.554)
Interpreting own patient data (0.546)
Undertaking technical midwifery procedures (0.503)

**Factor 2 (accounting for 5.5% of the variance, Cronbach's α = 0.8710): Extending service provision**
Identifying areas worthy of investigation in practice (0.747)
Applying pharmacology to practice (0.720)
Recognizing and managing risk in clinical care (0.699)
Requesting laboratory investigations and results (0.684)
Making appropriate patient referrals (0.617)
Liaising with other health care professionals (0.613)
Analysing patient data (0.610)
Designing systems for patient monitoring/observation (0.546)
Consulting with colleagues about care options (0.518)
Assessing patients' psychological and social needs (0.501)
Assessing costs and outcomes of procedures (0.497)

**Factor 3 (accounting for 3.9% of the variance, Cronbach's α = 0.6467): Assessment skills (0.488)**
Appraising own and other's performance (0.488)
Critically evaluating published research (0.373)

These factors fall into three clearly defined areas representing the core themes that constitute the midwife's role. These could inform the curriculum of initial midwife training, as well as job descriptions and appraisal frameworks.

### Occupational profile of midwives: subsample comparisons

#### By province

To establish whether there were any differences between the importance ratings given to each task by midwives in each of the three provinces, a series of one-way ANOVAS for unrelated designs was conducted, with post hoc Tukey tests where the differences were significant. Thirty-two differences between regions emerged, suggesting that perceptions of occupational roles differ by region. Because only three regions (out of 34 at the time of writing) were involved in the study, the details of the results are less relevant than that differences exist and that this methodology has the capacity to reveal them. It could therefore be used to inform both core training and job descriptions at a local, rather than national, level. The implications of this finding also need careful guidance by central policy to ensure standardization of quality practice across the country.

#### By work location (hospital versus community)

Comparisons of the criticality ratings awarded by hospital and community-based midwives were undertaken to establish whether the occupational profiles of these two subgroups differed. A series of unrelated t-tests was conducted on the importance ratings given to each of the 40 tasks; only four significant differences emerged. These results suggest that there is a 90% overlap between the occupational roles of hospital and community midwives.

#### By grade of midwife (D3 versus PPB/A)

Comparisons of performance ratings given to each task by D3 and PPB/A midwives were compared using a series of unrelated t-tests. Thirty-five of the 40 tasks were perceived to be more important by the D3 midwives than by the PPB/A midwives. However, it should be emphasized that all tasks received high criticality ratings (in excess of 5.60 in each case), and are therefore perceived to be important to all midwives in absolute terms, irrespective of grade. The results are presented in Table 2.

**Table 2:** Comparison of D3 and PPB/A midwives' importance ratings

**Task**	**t**	**P**	**Implication**
Establishing a relationship with patients	-3.245	0.001	More important for D3 midwives
Designing systems for patient monitoring/observation	-6.882	0.000	More important for D3 midwives
Applying pharmacology to practice	-4.706	0.000	More important for D3 midwives
Identifying areas worthy of investigation in your practice	-3.421	0.001	More important for D3 midwives
Liaising with other health care professionals	-4.013	0.000	More important for D3 midwives
Assessing costs and outcomes of procedures	-3.837	0.000	More important for D3 midwives
Analysing patient data	-5.175	0.000	More important for D3 midwives
Requesting laboratory investigations/results	-3.128	0.002	More important for D3 midwives
Assisting patients in making informed choices	-2.934	0.004	More important for D3 midwives
Assessing patients' physical needs	-3.028	0.003	More important for D3 midwives
Making appropriate patient referrals	-4.221	0.000	More important for D3 midwives
Appraising own and others' performance	-4.491	0.000	More important for D3 midwives
Consulting with colleagues about care options	-3.134	0.002	More important for D3 midwives
Critically evaluating published research	-3.786	0.000	More important for D3 midwives
Recognizing and managing risk in clinical care	-4.673	0.000	More important for D3 midwives
Working as a member of a team	-5.029	0.000	More important for D3 midwives
Assessing patients' psychological and social needs	-5.476	0.000	More important for D3 midwives
Prioritizing work according to patients' needs	-3.192	0.002	More important for D3 midwives
Undertaking technical midwifery procedures	-6.045	0.000	More important for D3 midwives
Undertaking health promotion/prevention activities	-2.919	0.004	More important for D3 midwives
Showing patients and their families how to do things	-3.239	0.002	More important for D3 midwives
Developing joint working arrangements with others	-2.498	0.014	More important for D3 midwives
Introducing new ideas into own clinical work	-7.261	0.000	More important for D3 midwives
Writing clinical, shift and other reports	-2.580	0.11	More important for D3 midwives
Getting on with colleagues	-2.782	0.006	More important for D3 midwives
Interpreting results from clinical investigations	-3.058	0.003	More important for D3 midwives
Collecting own patient/clinical and surveillance data	-3.650	0.000	More important for D3 midwives
Instructing/training students/junior members of staff	-3.451	0.001	More important for D3 midwives
Undertaking clinical examinations of patients	-3.116	0.002	More important for D3 midwives
Actively assisting in change management activities	-6.448	0.000	More important for D3 midwives
Interpreting own patient data	-4.636	0.000	More important for D3 midwives
Inputting data into written/computerized records	-4.730	0.000	More important for D3 midwives
Planning patients' discharge	-5.145	0.000	More important for D3 midwives
Locating/accessing relevant equipment for clinical work	-4.019	0.000	More important for D3 midwives

These results suggest that the new diploma training programmes are making a difference to occupational roles, and that it might be expedient to introduce different job descriptions for each grade, where applicable.

### Training needs

#### Whole sample

A comparison of the scores for ratings A and B on the questionnaire items was conducted using a series of related t-tests. This provided an index of training needs, which could be prioritized according to the significance of the need. The results indicated that for the whole sample, all 40 items demonstrated a significant training need at p < 0.0001. This suggests that the respondents perceived themselves to have skill deficits in all the areas covered.

It should be noted that significant differences between importance and performance can be recorded even if the importance score is low. In other words, a task that is not particularly crucial to the successful delivery of a respondent's role may still have a training need. However, 38 out of the 40 items all had importance ratings in excess of 6.00 (minimum 1.00, maximum 7.00), and the remaining two (assessing costs and outcomes of procedures; undertaking budget planning activities) had importance scores between 5.5 and 5.9. This suggests that all tasks were perceived to be crucial to the midwives' role, and therefore the training needs reported on each would need to be addressed if the skill deficits are to be ameliorated.

The training needs were factor-analysed according to standard procedures [7], in order to establish whether any coherent clusters of skill deficit existed that could be used to form the basis of continuing professional development modules. Using a Varimax rotation, three factors were produced, which together accounted for 38.5% of the variance (KMO test of sampling adequacy = 0.911, Bartlett's test of sphericity p = 0.000, and eigen values >1.0). These are presented in Table 3.

**Table 3:** Factor structure of training needs of midwives

**Factor 1 (accounting for 29.3% of the variance Cronbach's α = 0.8941): Clinical and service management**
Interpreting results from clinical investigations (0.700)
Undertaking clinical examinations (0.671)
Interpreting own patient data (0.644)
Making decisions about patients' clinical problems (0.617)
Introducing new ideas into clinical work (0.602)
Instructing/training students/junior staff (0.589)
Writing clinical/shift/other reports (0.580)
Inputting data into written/computerized records (0.569)
Undertaking budget planning activities (0.567)
Undertaking health promotion/prevention activities (0.566)
Collecting own patient/clinical/surveillance data (0.564)
Locating and accessing equipment (0.554)
Using technical equipment (0.547)
Prioritizing work according to patients' needs (0.526)
Planning/organizing patients' treatment (0.504)

**Factor 2 (accounting for 5.1% of the variance; Cronbach's α= 0.8678): Extending the service provision**
Making appropriate patient referrals (0.634)
Requesting laboratory investigations and results (0.597)
Assisting patients in making informed choices (0.530)
Analysing patient data (0.524)
Liaising with other health care professionals (0.509)

**Factor 3 (accounting for 4.1% of the variance, Cronbach's α= 0.4995): Assessment skills**
Critically evaluating published research (0.591)
Identifying areas worthy of investigation (0.583)
Assessing costs and outcomes of procedures (0.551)

#### By work location: hospital versus community midwives

Hospital and community-based midwives were compared to see if they demonstrated any difference in training needs. A series of unrelated t-tests was computed to compare the two groups on the difference scores between rating A and rating B, for each item. Four items yielded significant differences between the groups, thereby suggesting a significant (90%) level of overlap between the training needs of the two groups of midwives and indicating the need for a common CPD programme.

#### By grade of midwife

D3 midwives' responses were analysed to establish their training needs (n = 52). Comparisons of the importance and performance ratings for each of the 40 items were undertaken using a series of related t-tests. The results are presented in Table 4 in order of statistical significance. (All tasks had criticality ratings in excess of 6.00, meaning that each was considered to be important to the respondents' role.)

**Table 4:** Training needs of D3 midwives

**Significant at =< 0.001**
Applying pharmacology to practice
Identifying areas worthy of investigation
Analysing patient data
Requesting laboratory investigations
Assessing patients' physical needs
Appraising own and others' performance
Consulting with colleagues about care options
Critically evaluating published research
Assessing patients' psychological and social needs
Undertaking technical nursing procedures
Introducing new ideas into own clinical work
Actively assisting in change management activities
Interpreting own patient data
Planning patients' discharge

**Significant at 0.01 and less**

Designing systems for patient monitoring and observation
Liaising with other health care professionals
Assessing costs and outcomes of procedures
Working as a member of a team
Prioritizing work according to patients' needs
Undertaking health promotion activities

**Significant at 0.05 and less**

Recognizing and managing risk in clinical care
Planning and organizing patients' treatment
Using technical equipment
Developing joint work arrangements with others

The same analyses were undertaken for PPB/A midwives' training needs, using a series of related t-tests (n = 280). Significant training needs were demonstrated for all 40 tasks, with p < 0.001 in each case. Again, all tasks were rated at >6.0 in terms of importance, except "assessing costs and outcomes of procedures", "appraising own and others' performance", "critically evaluating published research", "undertaking budget planning activities", and "actively assisting in change management activities". Each of these had criticality ratings of between 5.5 and 5.9. This means that all tasks were considered to be highly salient to the role and therefore merit further training and development.

#### Training needs by province

Comparisons of midwives' training needs by province were computed using a series of one-way ANOVAS for unrelated designs. Significant ANOVA results were followed by post hoc Tukey tests There were seven significant differences, suggesting that the training needs of midwives working within different provinces are generally similar, enabling a common CPD programme to be put together. However, the significant differences indicated above suggest some variation in need, which might merit additional courses to be developed that would meet the needs of these subgroups. This methodology could be used to inform and customize CPD provision at a local level, as part of a continuing programme.

### Impact of having a job description

Because it might be expected that having a job description would provide a more informed perception of the nature of the occupational role, the criticality ratings (rating A) awarded by those midwives who had a formal job description (n = 174) were compared with those who did not (n = 158), using a series of unrelated t-tests. Three significant differences emerged, suggesting that having a formal job description made very little difference to how the midwives construed their jobs.

Similarly, having a clear job description might enable a clearer self-assessment of training needs; a series of unrelated t-tests was undertaken to compare the training needs (ratings A minus B) of those midwives with a job description and those without. Only two significant differences emerged, suggesting that having a job description makes only a marginal difference to how midwives construe either their occupational role or their training needs.

### Impact of having an individual performance assessment

The purpose of an IPA is to provide constructive feedback about current performance levels; it would be anticipated that midwives who had been through individual performance assessment would have a clearer idea both of how well they were discharging their role responsibilities and what their development needs were. To check this assumption, a series of unrelated t-tests was conducted to compare the performance ratings (rating B) of those midwives who received formal performance reviews (n = 243) with those who received no formal performance review (n = 89). No significant differences were found between the two groups, suggesting that having the Government Annual Performance Review makes no difference to how individual midwives evaluate their own performance.

To establish whether individual performance assessment made any difference to midwives' self-reported training needs, comparisons of the above groups were made for their training needs scores (ratings A minus B), using a series of unrelated t-tests. Only one significant difference emerged, suggesting that having the job review makes very little difference to how midwives perceive their own training needs.

## Discussion

The results obtained from this survey will be discussed in line with the subheadings used in the Results section above.

### Occupational profile of midwives: whole sample

All 40 tasks were rated as highly important (average score in excess of 5.6 out of a maximum 7.0), thereby suggesting that these tasks are highly relevant to the midwives' jobs and therefore could constitute the foundation topics in the basic midwifery training. While these may not be an exhaustive list of relevant clinical tasks, they offer a set of core competences on which to base a curriculum, job description and performance appraisal; these components could also be modified to meet local needs.

To ascertain whether these tasks fell into coherent clusters of skills that could inform a framework for the basic midwifery education curriculum, an exploratory factor analysis revealed three factors, the first two of which were highly internally reliable. The third factor fell just below the 0.70 level, which may be attributable to the fact that only two variables were loading on the final factor (Table 1).

The first factor represented clinical and service management tasks; (broadly encompassing factors 1 and 3 from paper 1 [6]) the 18 items loading on this factor fall into two clear topics – the management and administration of general service practicalities, such as undertaking budget planning activities and writing clinical/shift reports; and management of more direct clinical and patient focussed topics, such as planning patients' discharge and undertaking health promotion/prevention activities. If this factor were used as a logical basis for curriculum development, then the natural split in the items could inform two separate modules – the management and administration of general service activities and the management of patient care.

The second factor, provisionally labelled "Extending Service Provision" reflects the need to work effectively with other providers in secondary and tertiary care. Given that a large proportion of clinical provision in Indonesia is made through the primary care sector, there is a clear need to liaise with other service providers in order to manage the midwifery pathways effectively; this factor, then, has logical appeal.

The final factor ("Developing Own and Others' Practice"), comprising two variables, may be considered to be part of a wider reflective practice issue, and, as such, could be expanded and developed to form a module in basic training. Factors 2 and 3 map onto Factors 5 and 6 from paper 1 [6]. While reflective practice in midwifery is a new concept in Indonesia, a number of recently introduced mechanisms in the service are encouraging this development, such as perinatal audit, peer review and the reflective case discussions recently introduced following a preliminary analysis [5] of these findings.

The salience of all tasks and their clustering into coherent factors could inform national standards of training and accreditation of courses. This would have the effect of ensuring that all qualifying midwives possessed the core competences, ultimately enhancing training and midwifery provision.

Moreover, these criteria could be used as outline job descriptions, potentially ensuring that service delivery neither falls short of expected standards nor exceeds the boundaries of professional expertise. In a country where there has been a history of nurses and midwives substituting for each other, often in highly specialized clinical areas such as deliveries, clear local job descriptions should go some way towards effecting more clearly defined professional roles. Job descriptions also provide a set of guideline activities against which to monitor performance.

Given that the Indonesian government has made annual performance assessment compulsory for all public service workers, a detailed job description would provide a clear benchmark against which to conduct these reviews. These are also important to be able to judge continuous quality improvement initiatives.

### Occupational profile of midwives: subgroup comparisons

#### By province and by workplace: hospital versus community

While the above listing of salient skills provides a useful foundation for the education and development of midwives, there are variations in how midwifery services are discharged, largely as a function of geopolitical factors. The diversity of terrain makes health services difficult to access for many rural communities, there may be limited facilities in remote areas and the distinct environments of community versus hospital-based midwifery provision together may mean that midwifery care varies in nature and quality across provinces and care settings.

It might therefore be expected that midwives working in a variety of situations might view their roles differently. However, the current findings suggest very few differences between hospital and community midwives in this regard (90% overlap in role function). In contrast, there were distinctions between midwives working in North Sulawesi, East Kalimantan and North Sumatra, with 32/40 tasks being attributed with varying levels of importance for each province. The results clearly indicate that the demands on the role of the midwife varies by province and that job role descriptions could be usefully adjusted to take account of these variations.

However, as only three of the 34 provinces were sampled in this survey, the detailed differences may of less relevance than that significant differences existed. This would highlight the need for a more systematic exploration of midwives' roles across all provinces, in order that any customization of the basic pre-service national curriculum could be pursued at the province level. It would also facilitate the development of specific job descriptions and performance review criteria, to reflect the diversity of functions.

#### By grade of midwife

Comparisons of importance ratings given by different grades of midwives indicated that D3 midwives perceived 35 of the 40 tasks to be more important than did PPB/A midwives (Table 2). While it should be emphasized that in absolute terms all midwives rated every task as being important (in excess of 5.60), the higher values attributed by the D3 midwives might reflect the higher level of performance expected of this more highly educated grade and offer confirmation that new training programmes are having an impact on how midwives at various grades conceptualize their jobs.

### Training needs

#### Whole sample

Comparisons of the importance and performance ratings provided an index of training need [8]; these can be computed for any subsample as well as for the whole sample, thereby offering information about the education and development needs of specific groups of midwives. Calculations for the whole sample indicated that there was a highly significant training need for all 40 tasks, thereby indicating a self-perceived skill deficit across the spectrum of activities. As all tasks were also given high importance ratings (see above), this means that it is not possible to prioritize the training needs either by salience of task or by significance of the importance/performance gap. Therefore, taken as a whole, all 40 items need to be addressed in a continuing professional development programme.

To provide a rational framework for CPD provision, a factor analysis was undertaken of the training needs data. This produced three factors that were broadly equivalent to those that emerged from the factor analysis of the criticality ratings; they have therefore been given similar labels (Table 3). While the overlap of items loading onto each factor is not identical, the underlying content is similar, and confirms that the subgroups of tasks that constitute the midwife's job all require further professional development. These factors could easily form the basis of discrete, stand-alone modules in a CPD programme, which might include service management, extending midwifery service provision and assessment skills.

#### Training needs: subgroup comparisons

As the midwife's role and function in Indonesia is diverse, it would seem reasonable to suppose that their development needs might differ according to the nature of their role. Any differences that emerged in terms of training need could be used to inform customized training packages, which would optimize the use of limited education budgets while maximizing the benefit to the midwife. The comparisons that were undertaken demonstrated that there were only four differences between the hospital and community midwives' training needs, which suggests that, as with the job description analysis, the work setting does not significantly affect the nature of CPD needs.

#### Training needs by grade of midwife

Analysis of training needs by grade of midwife suggested that the D3 midwives (diploma level) had 24 training needs that could be prioritized by significance (Table 4), while the PPB/A midwives recorded highly significant training needs on all 40 tasks (p < 0.001). The fact that the D3 midwives demonstrated fewer training needs, and that these were at a variety of levels, may reflect that this subgroup has been educated to a higher level, thereby obviating the need for training across all tasks.

#### Training needs by province

Comparisons of training needs by province produced only seven differences, with midwives in North Sumatra having fewer reported training needs than those from East Kalimantan and North Sulawesi. While these minor differences could be used to inform specialized CPD packages, the similarity of training needs across all subsamples might support a standardized set of modules that could be delivered to all midwives, without serious loss of specificity or additional expenditure incurred by setting up separate courses. It should be noted, though, that only three provinces were sampled. More extensive surveys would be needed to confirm whether CPD packages should be customized, or whether a more generic package of training would suffice across all regions.

#### Impact of having a job description

The impact of having no job description is potentially serious, in that without role boundaries, and with pressure resulting from understaffing, it is common for midwives to undertake tasks beyond their professional competence levels [5]. Not only does this compromise the midwife's professionalism, but it could also negatively affect patient well-being. It might therefore be expected that those midwives who had formalized job descriptions would have a clearer set of expectations about the nature of their job and where their development needs lay.

The 174 midwives who had a formal job description were compared with the 158 who did not, on importance ratings and training needs. Very few differences emerged from either of these comparisons, thereby suggesting that a clear definition of occupational role makes little difference to how midwives perceive their jobs. However, it is worth noting that after this study, field assessments in more than 30 districts have shown that where there is a job description, it is inadequate [5] and therefore would be unlikely to have affected the perceptions of the midwives. Moreover, role conceptualization might also be framed by necessity: where there is a shortage of skilled health care professionals, it is conceivable that the midwife's role is determined by the demands placed on it, rather than by the skills described by a formal role outline. Some support for this contention comes from the fact that 51.5% (171) of the midwives surveyed undertook cleaning activities – a low-skill task, not commensurate with their educational or competence levels – possibly because of necessity, the legacy of traditional outdated practices and poor understanding of their patient-focused role.

#### Impact of the individual performance assessment

The requirement to have an annual individual performance assessment has been stipulated by the Indonesian government, as one mechanism by which to rate performance of staff and, from the results, to determine reward level. Without a regular and structured clinical performance review and feedback opportunity, it may be difficult for many midwives to recognize, reflect and improve on the quality of their clinical practice and service delivery, or to identify their future development needs. The present study indicates that this process does not appear to have any impact on midwives' perception of their current performance levels or training needs.

Comparisons of the 243 midwives who had undergone individual performance assessments and the 89 who had not, on both self-reported performance ratings (rating B), and training needs, revealed no significant differences between the two groups in terms of reported performance, and only one significant difference in training need. This suggests that the current method of conducting government performance assessments on midwives has no impact either on how midwives assess their own performance or their development needs. One explanation for this may be the relatively unstructured way in which individual performance assessments are currently conducted [5], with many midwives not knowing whether or not they have been appraised. Without a planned, methodical approach to professional development, it may be difficult for midwives to undertake useful reflection on their practice within an agreed skill framework.

#### Comparisons of nurses and midwives on occupational roles and training needs

There were areas both of similarity and difference between the nurses and midwives, which can best be summarized as follows:

• Both nurses and midwives perceived all the tasks to be very important to their work and therefore these all occupational functions are crucial across both samples.

• The occupational tasks clustered together in similar ways for both nurses and midwives, with minor differences in the task content of the clusters and their significance.

• Both nurses and midwives reported very little difference between their hospital and community roles.

• There was considerably more variation in the occupational role of the midwife across provinces than there was in the occupational role of the nurse.

• There was more variation in occupational role by grade of midwife than there was for grade of nurse.

• Both midwives and nurses have similar, high levels of training needs, which cluster together in comparable groups of tasks.

• There was more variation in the training needs of hospital and community nurses than there was between hospital and community midwives.

• There were more differences in training need between grades of midwife than between grades of nurse.

• There were fewer differences in training needs across provinces for midwives than for nurses.

• There was very little impact of having either a job description or individual performance reviews on the training needs of both nurses and midwives.

## Conclusion

The methodology and findings in this paper emphasize the need for a systematic approach to the pre- and postbasic education of midwives, in order to link provision with need. As noted in the related paper, some of the results from this study have already had a major impact on the development of quality control in the nursing and midwifery service in Indonesia, through the design of a system called a "Clinical Performance Development and Management System for Nurses and Midwives in Hospitals and Community".

This system has been tested and implemented in hospitals and health centres in 32 districts in nine provinces in Indonesia (brief details of the roll-out of the programme have been documented in the related paper). Preliminary evaluations in three provinces in 2003 through regular monitoring reviews show a clear focus on standards, on the roles outlined in job descriptions, increased motivation and enquiry [9].

The system is now being introduced through a training-of-trainers process and has been rolled out to many more districts in 2004. The system includes the dissemination of midwifery standards directly to the providers, locally developed job descriptions, a clear performance indicator-based monitoring system and group reflective case discussions. Initial findings were also taken into consideration with the review of the Midwifery Diploma Curriculum in 2002. The findings, together with those of the In-Service Evaluation [10], could make a substantial contribution to improvements in pre-service and in-service midwifery education in Indonesia.

The benefits of the Clinical Performance Development and Management System have been documented elsewhere [11], but can be summarized as both an enhanced understanding of management practices and the professional responsibility for patient care; service delivery that is commensurate with nurses' and midwives' training; changes in clinical practice through reflection, guidelines and case discussion; a link between performance and reward; and improvements in clinical, patient attendance and levels of patient complaints.

The development of the programme, its monitoring and evaluation were funded exclusively through WHO, with the World Bank, Asian Development Bank and local governments covering the cost of implementation. The full economic costs have yet to be evaluated, but there is little doubt that the implications of these studies have been both positive and considerable for health care provision in Indonesia.

## Competing interests

The author(s) declare that they have no competing interests.

## Authors' contributions

DH designed and managed the survey and organized its data collection and preliminary analysis, assisted by AH and YK; CH did a secondary data analysis and wrote the article. All authors read and approved the final article.
